# Responsiveness of the new index muscular echotexture in women with metastatic breast cancer: an exercise intervention study

**DOI:** 10.1038/s41598-022-19532-7

**Published:** 2022-09-07

**Authors:** Adrian Escriche-Escuder, Manuel Trinidad-Fernández, Bella Pajares, Marcos Iglesias-Campos, Emilio Alba, José Manuel García-Almeida, Cristina Roldán-Jiménez, Antonio I. Cuesta-Vargas

**Affiliations:** 1 Department of Physiotherapy, University of Malaga, C/ Arquitecto Peñalosa, 3, 29071 Málaga, Spain; 2 Instituto de Investigación Biomédica de Málaga (IBIMA), Málaga, Spain; 3 UGCI Oncología Médica Hospitales Universitarios Regional y Virgen de la Victoria, Málaga, Spain; 4 UGCI Endocrinología y Nutrición, Hospital Regional Universitario y Virgen de la Victoria, Málaga, Spain; 5 School of Clinical Sciences, Faculty of Health, Queensland University of Technology, Brisbane, QLD Australia

**Keywords:** Cancer, Biomarkers, Medical research, Oncology

## Abstract

Ultrasound imaging texture analyses may provide information on tissue homogeneity changes in metastatic breast cancer (MBC) through second-order analyzes based on the gray-level co-occurrence matrix. This study aimed to analyze the responsiveness and correlations of biomarkers of muscular and fat echotexture after an exercise intervention in women with MBC. A 12-week exercise intervention was conducted in 2019, including aerobic and strength training. Echotexture variables were obtained at baseline and after intervention from the quadriceps (Q) and biceps brachii and brachialis. Mean differences were calculated using the T-Student parametric test for dependent samples of the differences in the means (*P* = 0.05; 95% CI). Data obtained from 13 MBC women showed significant differences in some echotexture variables after the intervention. QLQ-BR23 questionnaire correlated with several echotexture variables from muscle and subcutaneous fat. PFS-R scale correlated positively with the Q Subcutaneous Fat Non-Contraction Homogeneity (R = 0.43, *P* < 0.05). Q Muscle Non-Contraction Energy and Q Muscle Non-Contraction Textural Correlation explained 90% of the variance of QLQ-BR23. Some muscle and subcutaneous fat echotexture biomarkers showed good responsiveness after the exercise intervention. Additionally, some muscle and subcutaneous fat variables correlated with QLQ-BR23 and cancer-related fatigue measured by PFS-R scale in MBC patients.

Trial registration: NCT03879096

## Introduction

Although medical advances have increased breast cancer (BC) survival by approximately 40% in the last three decades^1^, an increase in the incidence of metastatic disease has been observed. Thus, while an initial presentation of metastatic breast cancer (MBC) is not common, up to 30% of those diagnosed with BC at an early stage develop metastases in the following months^2^. Metastatic disease is currently considered incurable, and it is, along with recurrence-related complications, the most common cause of cancer-related deaths^3–5^. In this context, current treatments are aimed at delaying the progression of the disease, limiting its effects and maintaining the quality of life (QoL)^2^.

Many of the effects present in cancer disease are also found in MBC, but usually in a more severe way^6^. This is due both to the disease itself and to greater side effects of the treatments used in its management. Thus, the appearance of cancer-related fatigue (CRF), poor levels of functional capacity, lymphedema, peripheral neuropathy or pain is common, affecting QoL^7–10^. Changes in body composition are also frequent in women with BC, in muscle, in the form of sarcopenia, and fat tissue^11^. Sarcopenia, defined as the presence of low muscle mass and strength^12^, has been associated with greater toxicity from chemotherapy^13^, a greater decrease in functional capacity and strength^8,11,14^, and a worse prognosis^13,15^.

International guidelines suggest exercising and avoiding inactivity in patients with metastatic disease^16,17^. Different reviews support its safety in patients with cancer^18^, inducing benefits in functional capacity^19^, and patient-reported outcomes (PRO) such as CRF and QoL^8,20,21^, related to an improvement in architecture and body composition^22,23^. In addition, these interventions are effective both in the post-treatment period and in patients undergoing treatment, showing relevant differences with those groups that do not exercise^24^.

Clinicians and researchers have used different tools to allow adequate monitoring of the changes produced by the progress of the disease or treatment. Imaging techniques have proven to be useful for analysing sarcopenia and body composition^25^. Specifically, ultrasound imaging (US) tools are commonly used in clinical practice to offer reliable information about tissue architecture and composition through an affordable and non-invasive method^26–28^.

Among the US variables, thickness and echointensity (EI) have predominated for their ease of calculation. However, texture analyses in US, known as echotexture variables, have also been described in different diseases and healthy people^29–31^. In this sense, some authors have previously described a first-order variable that could better detect body composition and correlate with clinical variables, defined as echovariation^32^. This new echotexture variable, which provides information on tissue homogeneity^33^, can be analyzed through second-order analyzes based on the gray-level co-occurrence matrix (GLCM), considering pixels as pairs and analyzing the relative position of the patterns of grays in the image^34^. This new analysis has shown in other populations a capacity for discrimination similar to the echovariation, providing additional information on gray patterns^28^. However, its utility has not been studied in patients with MBC.

The study of echotexture variables could help to detect new biomarkers for detecting structural muscle and fat changes that allow monitoring the disease´s progress in MBC. However, this method has not been previously studied in this population.

### Objective

The aim of this study was to analyze the responsiveness and correlations of biomarkers of muscular and fat echotexture and PRO (CRF and QoL) after an exercise intervention in women with MBC. Authors hypothesized that some muscular and fat biomarkers measured by US (i) will have a good responsiveness after the exercise intervention; (II) will correlate with some PRO; (III) will explain changes in PRO after intervention.

## Methods

### Study design and setting

In 2019, an exercise intervention study was conducted in the Medical Oncology Unit at the University Clinical Hospital Virgen de la Victoria of Malaga (Spain).

The study was reported following the CONSORT checklist to ensure transparent content. The study was registered in ClinicalTrials.gov (NCT03879096) on 18/03/2019 prior to enrollment of participants, and was authorized by the Portal de Ética de la Investigación Biomédica de Andalucía Ethics Committee (2804/2016). All subjects signed written informed consent after being informed about the purpose and procedures of the investigation. This study adhered to the principles of the Declaration of Helsinki.

### Participants

Potentially eligible women with MBC were recruited by medical oncologists from the University Clinical Hospital Virgen de la Victoria (Malaga, Spain).

### Selection criteria

We included women older than 18 years, with a current diagnosis of metastatic breast cancer, not amenable to curative treatment.

Exclusion criteria were: (1) Cardiovascular event during the year prior to recruitment, including angor; cardiac rhythm disorders; acute pulmonary edema; or syncope of an unknown cause.

Women undergoing treatment (chemotherapy, radiotherapy, or hormone or monoclonal antibody treatment) were allowed to participate.

### Intervention

A previously described 12-week therapeutic exercise program^35^ was performed in groups, including aerobic exercise and strength training led by a physical therapist. The program aimed to generate neuromuscular and cardiovascular adaptations, and took into account the current recommendations in the oncology field^20^. Before the start of the intervention, an individual assessment of the functional capacity was done to assess individual restrictions and needs. The intervention included 30 min of strength training and 20 min of aerobic exercise.

### Measurements

At the beginning of the study, a first evaluation session was carried out to collect information on demographic and anthropometric variables, and conductquestionnaires, imaging and functional capacity tests. After a 12-week intervention, the evaluation session was conducted again to assess the presence of changes.

### Variables

#### Descriptive outcomes

Anthropometric and demographic data (age, height, weight, and body mass index), as well as medical patient-reported outcomes (PRO) of the participants (years since cancer diagnosis, months since metastasis diagnosis, affected breast side, modality of surgical intervention, metastatic disease site, previous oncologic treatment and current treatment, cardiovascular risk factors, and comorbidities), were collected.

#### PRO

##### Piper fatigue scale-revised (PFS-R)

The PFS-R scale is commonly used to assess CRF. This multidimensional fatigue measure contains 22 items scoring from 0 to 10. A revised version was used for this study, converting the total score (from 0 to 220) to a scale from 0 to 10. The interpretation of the results for the presence of fatigue are: 0, none; 1–3, mild, 4–6, moderate; 7–10, severe)^36^ High reliability (Cronbach’s α = 0.96) has been previously found in people with cancer of the Spanish version applied in this study^37^.

##### European organization for research and treatment of cancer breast cancer-specific quality of life questionnaire (QLQ-BR23)

The QLQ-BR23 assesses the QoL using a 4-point scale of 23 items. Each item can be scored from 1 to 4 (from not at all to very much, respectively), and the resulting score can be linearly converted to a 100-point scale. This questionnaire has previously shown acceptable reliability (Cronbach’s α = 0.46–0.94)^38^.

#### US outcomes

**Figure 1:**
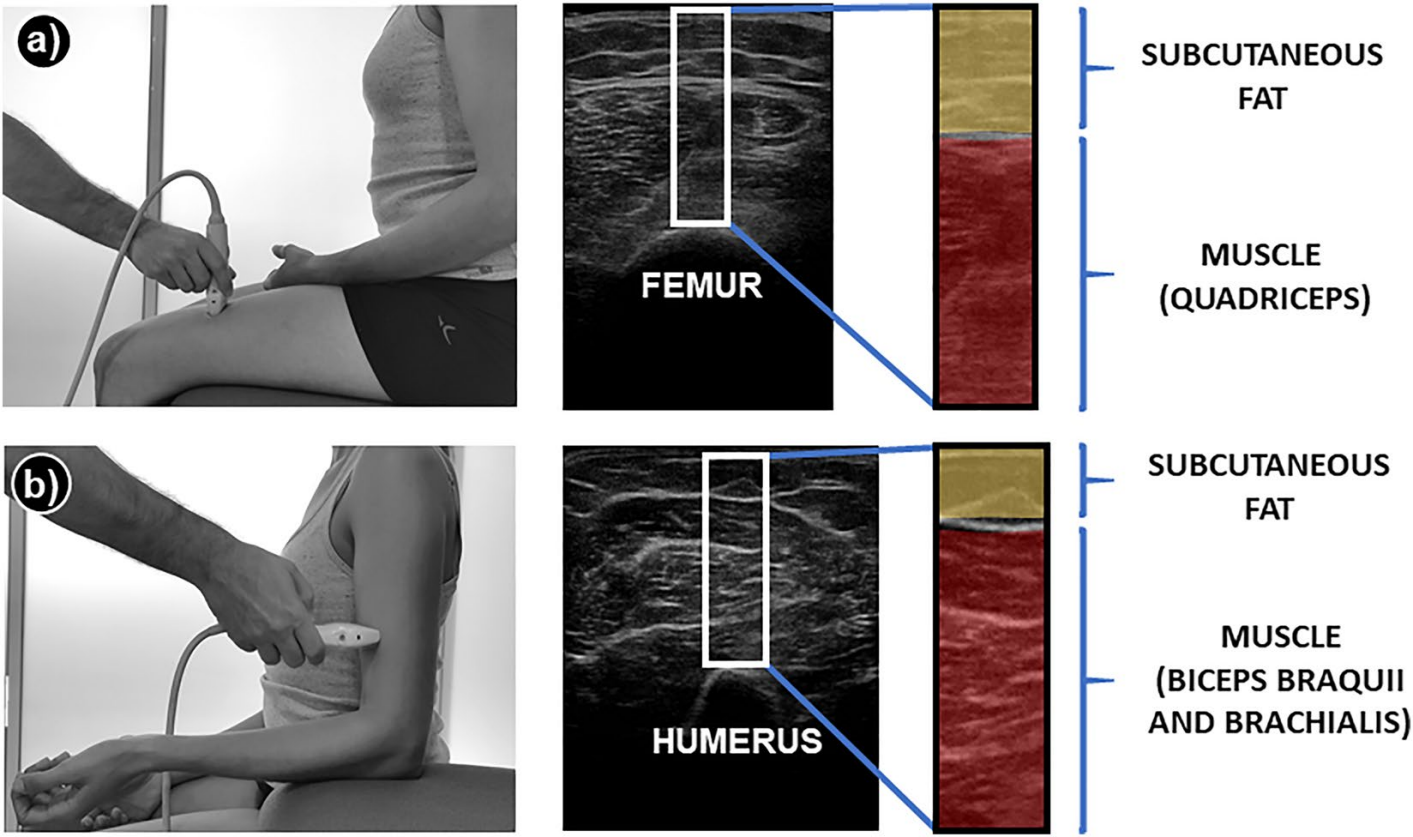
Position of the patient and ultrasound probe and range of interest (white rectangle) of sample images (**a**: quadriceps; **b**: biceps/brachialis).

For US, the 2D ESAOTE MyLab25Gold (Esaote SpA, Genova, Italia) US device was used before and after the interventionA 5 cm linear array transducer, with a standard gain of 70% and a frequency of 12 Hz was used. The depth was adapted to the anthropometric characteristics of each subject to allow an adequate framing of the target structures in the image capture. The patient was seated on an examination table, while an expert operator stood in front of the subject, holding the US transducer with one hand and fixing the patient's hand or leg with the other hand. In all participants, the image of the thigh (15 cm from the upper pole of the patella)^39^ was taken first and then the image of the arm (at the mid-point and anterior part of the humerus)^40^ was captured (Fig. 1). In addition, at both locations, a non-contraction image was captured first and then during a 5-s isometric muscular contraction was taken a second image capture. Transducer was always placed transversely to the direction of the fibers.

The captured images were exported to bmp files (resolution of 800 × 652 pixels and 96 dpi), and were processed offline using a code of MATLAB (Version R2018b, MathWorks, Natick, USA), as detailed in the section below.

From the images extracted from the arm and leg, a range of interest (ROI, maximum interest zone) of 1 cm in length on the horizontal axis was selected, in which the muscle tissue and the subcutaneous fat tissue were identified. The echotexture analysis, based on the analysis of the GLCM, was conducted for each of these selections. This analysis is derived from the studying the angular relationship between the contiguous pixels, and its results have no unit. Five echotexture variables were selected^28^:

- *Homogeneity:* also known as inverse difference moment (IDM), this parameter is associated with the local homogeneity of the pixels. If the ROI is homogeneous, the homogeneity have a higher value.
- *Entropy:* the entropy parameter is inversely related with homogeneity. When the homogeneity of the ROI is high, a lower entropy is obtained.
- *Energy* also known as angular second moment, is related to the ROI’s homogeneity. When the area is homogeneous, the energy value is also high.
- *Contrast:* the contrast assesses the variation between the contiguous pixels. When there is a high variation, the contrast is also high.
- *Textural correlation:* for those ROIs in which there are regions with similar levels of gray, the value of the textural correlation is higher.

Quadriceps (Q) and Biceps brachii and Brachialis (BB) areas were analyzed using echotexture variables. In each area (Q and BB), an image capture was made in a Non-Contraction situation and then analysedduring muscle Contraction, analyzing Muscle and Subcutaneous Fat parameters. These measurements were carried out at baseline (before intervention) and after the 12-week exercise program (after intervention). The combination of the above described five variables in the different locations (Q and BB), situations (Contraction and Non-Contraction), analyzed tissue (Muscle and Subcutaneous Fat), and time (before and after intervention), together with the variables obtained from the difference between the Contraction and Non-contraction situations (Difference), provides 30 variables for each location.

### US images processing and analysis

The images were processed using a script designed for this project using MATLAB (Version R2018b, MathWorks, Natick, USA). This Matlab code allows researchers to manually select the ROI in the image in a standardized way to make different images comparable. The characteristics of the ROI were a length of 1 cm on the horizontal axis and a distance on the vertical axis from the bone (upper pixel of the femur or humerus) to the superficial layer of the skin (upper pixel of the image). In all the images, the ROI was centered with the midline of the bone. Once the ROI is obtained, the operator can manually select the muscle (area between bone and fascia) and the subcutaneous fat (between fascia and superficial skin) areas. An example of ROI and areas selection in the thigh and arm is shown in Fig. 1. Using this selection, the script converts the image to a gray scale and gets the previously described echotexture variables.

### Statistical methods

Analyzes were conducted using SPSS for Windows (version 25.0, SPSS Inc., Chicago, IL, USA). The analyzes were performed using the two measurements of the study, baseline (pre) and after 12 weeks (post). Mean and standard deviation (SD) of quantitative variables were calculated. Absolute frequencies and percentages were used to describe qualitative variables. The Kolmogorov–Smirnov test was used to determine the normal distribution of the variables. In order to obtain the mean intragroup differences from baseline to the post-measurement, T-Student parametric test for independent samples was conducted. *P*-values < 0.05 were accepted as statistically significant, establishing a 95% confidence interval.

Inferential statistics were carried out between echotexture and PRO outcomes with all before and after intervention measurements, using the Pearson r correlation coefficient. The correlation was described as: poor (r < 0.49), moderate (r = 0.50–0.74), or strong (r > 0.75)^41^. Results with a *P* value < 0.05 were considered statistically significant. Additionally, a linear regression analysis was performed with those PRO with significant correlations with echotexture outcomes to retrieve the best regression model.

### Bias

In order to reduce execution-related bias, an expert and blinded researcher conducted the data analysis.

## Results

### Participants

Thirteen women with MBC voluntarily participated in the prospective study. Subjects’ baseline descriptive, medical and oncological characteristics are shown in Table 1.

**Table 1:** Participant descriptive, medical and oncological variables (n = 13).

	Mean (SD)	Min–Max
Age (years)	48.69 (7.20)	40–59
Weight (kg)	74.78 (17.90)	51–119.50
Height (m)	1.63 (0.06)	1.57–1.79
BMI (kg/m2)	27.12 (5.76)	19.84–36.72
Time since diagnosis (in years)	2.67 (0.81)	1.10–3.00
Time since metastasis diagnosis (in months)	26.25 (36.02)	0.50–180.00

	Outcome	Value (%)
Surgery intervention	Mastectomy	7 (53.85%)
	Breast-conserving	2 (15.38%)
	None	4 (30.77%)
Systemic treatment	Chemotherapy	13 (100.00%)
	Hormone therapy	11 (84.62%)
	Monoclonal antibody	6 (46.15%)
	Radiotherapy	4 (30.77%)
Metastatic disease site	Visceral (liver, lung or CNS)	13 (100.00%)
	Non-visceral	10 (76.92%)
	Visceral and Non-visceral	10 (76.92%)
Bone metastases	Subjects with bone metastases	10 (76.92%)
	Spine	8 (61.48%)
	Femur	5 (38.46%)
	Pelvis	5 (38.46%)
	Thorax	5 (15.38%)
	Humerus	2 (38.46%)
Type of bone metastases	Mixed	5 (38.46%)
	Osteoblast	4 (30.77%)
	Osteolytic	1 (7.69%)
Current treatment	Chemotherapy	4 (30.77%)
	Hormone therapy	8 (61.48%)
	Monoclonal antibody	4 (30.77%)
	Radiotherapy	4 (30.77%)

*BMI* body mass index, *CNS* central nervous system.

### Intragroup differences

After 12 weeks of intervention, Q Muscle Non-Contraction Contrast (1534.59394, *P* = 0.04) and Q Subcutaneous Fat Non-Contraction Entropy (− 0.17057, *P* = 0.04) showed statistically significant intra-group changes (Table 2). In the area of BB, only the Difference of Homogeneity between non-contraction and contraction images of the Subcutaneous Fat revealed a statistically significant decrease (− 0.00917, *P* = 0.01). The results in the rest of the BB variables are shown in Table 3.

**Table 2:** Textural analysis in quadriceps during the pre and post intervention (n = 13).

	Variables	Pre (mean)	SD	Post (mean)	SD	Mean difference
**Muscle**						
Non-contraction	Contrast	8757.04	5088.92395	7222.4508	3560.04735	− 1534.59394*
	Textural Correlation	**− **.0034	.01151	**− **.0020	.03128	.00140
	Energy	.0001	.00007	.0001	.00005	**− **.00001
	Homogeneity	.0441	.01039	.0456	.00956	.00151
	Entropy	6.2275	.50920	6.3964	.39976	.16886
Contraction	Contrast	10,423.0381	7587.56771	11,998.0654	8213.12988	1575.02724
	Textural Correlation	**− **.0005	.00408	**− **.0015	.00293	**− **.00100
	Energy	.0001	.00006	.0001	.00005	**− **.00002
	Homogeneity	.0447	.01249	.0416	.01266	**− **.00309
	Entropy	6.2823	.42038	6.4092	.24595	.12684
Difference	Contrast	1665.9933	3956.43316	4775.6145	7786.70822	3109.62119
	Textural Correlation	.0029	.01124	.0005	.03189	**− **.00239
	Energy	.0000	.00001	.0000	.00003	**− **.00000
	Homogeneity	.0006	.00762	**− **.0040	.00833	**− **.00460
	Entropy	.0548	.21564	.0128	.43280	**− **.04202
**Subcutaneus fat**						
Non**-**contraction	Contrast	2944.9681	1831.61860	3176.6703	2666.02089	231.70219
	Textural Correlation	**− **.0037	.02196	.0011	.01901	.00479
	Energy	.0002	.00009	.0001	.00008	**− **.00001
	Homogeneity	.0659	.01801	.0688	.01994	.00294
	Entropy	6.2549	.43994	6.4255	.35950	.17057*
Contraction	Contrast	2001.3163	1348.38267	2175.8404	1667.20386	**− **594.70125
	Textural Correlation	.0008	.01290	**− **.0035	.00711	**− **.00408
	Energy	.0002	.00012	.0002	.00010	**− **.00004
	Homogeneity	.0748	.01591	.0739	.01729	**− **.00652
	Entropy	6.4242	.30575	6.4444	.41412	.14277
Difference	Contrast	**− **943.6518	807.18802	**− **1000.8299	1458.75470	**− **57.17816
	Textural Correlation	.0046	.02601	**− **.0045	.02245	**− **.00910
	Energy	.0000	.00005	.0000	.00003	**− **.00001
	Homogeneity	.0089	.01034	.0051	.00844	**− **.00387
	Entropy	.1693	.26325	.0190	.24313	**− **.15034

* P < 0.05.

**Table 3:** Textural analysis in biceps braquii and brachialis during the pre and post intervention (n = 13).

	Variables	Pre (mean)	SD	Post (mean)	SD	Mean difference
**Muscle**						
Non**-**contraction	Contrast	9298.8469	4659.72209	13,107.5241	7018.34537	3808.67722
	Textural Correlation	**− **.0024	.00891	**− **.0004	.00705	.00203
	Energy	.0001	.00005	.0001	.00006	**− **.00000
	Homogeneity	.0424	.01016	.0393	.01463	**− **.00308
	Entropy	6.1899	.28386	6.3095	.40122	.11968
Contraction	Contrast	14,262.9518	9608.99989	13,226.0699	6162.11902	**− **1036.88193
	Textural Correlation	.0026	.01629	**− **.0004	.00580	**− **.00302
	Energy	.0001	.00004	**− **.0011	.00225	**− **.00113
	Homogeneity	.0366	.00844	.0367	.00849	.00004
	Entropy	6.2902	.33165	6.3488	.41499	.05853
Difference	Contrast	4964.1049	8668.16840	118.5458	6068.91523	**− **4845.55914
	Textural Correlation	.0050	.01760	**− **.0001	.00774	**− **.00505
	Energy	.0000	.00001	**− **.0011	.00226	**− **.00113
	Homogeneity	**− **.0057	.01187	**− **.0026	.00948	.00312
	Entropy	.1004	.32487	.0392	.50906	**− **.06115
**Subcutaneus fat**						
Non-contraction	Contrast	2221.0160	2923.72719	1380.9936	728.34674	**− **840.02237
	Textural Correlation	.0025	.00855	**− **.0034	.01475	**− **.00587
	Energy	.0004	.00034	.0003	.00023	**− **.00003
	Homogeneity	.0822	.02333	.0844	.01628	.00214
	Entropy	6.3377	.39196	6.1016	.54329	**− **.23614
Contraction	Contrast	1380.3382	693.48684	1838.2430	1679.82847	457.90478
	Textural Correlation	.0028	.00520	**− **.0063	.01581	**− **.00914
	Energy	.0005	.00031	.0006	.00099	.00011
	Homogeneity	.0856	.01689	.0786	.01543	**− **.00703
	Entropy	6.3283	.47152	6.0615	.71016	**− **.26677
Difference	Contrast	**− **840.6778	2439.63708	457.2494	1490.03081	1297.92715
	Textural Correlation	.0003	.00810	**− **.0029	.01528	**− **.00327
	Energy	.0001	.00023	.0002	.00085	.00014
	Homogeneity	.0034	.00904	**− **.0058	.01201	− .00917*
	Entropy	**− **.0094	.35164	**− **.0400	.52371	**− **.03064

^*^P < 0.05.

The intragroup analysis of the questionnaires revealed slight changes between the initial measurement and the measurement after 12 weeks. The QLQ-BR23 showed a small decrease from baseline (40.73 ± 11.16) to post-measurement (39.45 ± 11.59). The PFS-R scale decreased slightly from baseline (3.70 ± 2.86) to post (3.45 ± 1.78). However, none of these changes made a statistically significant difference.

### Correlations

In the analysis of the correlations between the US variables and the questionnaires, the QLQ-BR23 questionnaire correlated positively with the Q Muscle Non-Contraction Energy (R = 0.56, *P* < 0.01), the Q Muscle Contraction Energy (R = 0.45, *P* < 0.05), and the BB Fat Contraction Homogeneity (R = 0.50, *P* < 0.05), while correlated negatively with the Q Muscle Non-Contraction Entropy (R = − 0.49, *P* < 0.05) and the Q Muscle Contraction Textural Correlation (R = − 0.41, *P* < 0.05). Only the Q Subcutaneous Fat Non-Contraction Homogeneity for the PFS-R scale showed a positive correlation (R = 0.43, *P* < 0.05).

### Regression analysis

Different regression models were explored that allowed explaining, using US echotexture variables, changes produced in the subjective variables studied through the questionnaires. Table 4 shows the best model found in the linear regression analysis. Multiple linear regression analysis showed that Q Muscle Non-Conn Energy and the Q Muscle Non-Contraction Textural Correlation, after adjusting by age and weight explained 90% of QLQ-BR23 variance (R^2^ = 0.90, *P* < 0.01).

**Table 4:** Multiple regression analysis about the Quality of Life Questionnaire Breast Cancer (QLQ-BR23).

Dependent variables	Predictor variables	Standardized β	R	R^2^
QLQ-BR23	Q Muscle Non-Contraction Energy	0.63**	0.95	0.90**
	Q Muscle Non-Contraction Textural Correlation	− 0.35**		
	Age	0.24		
	Weight	0.80**		

*QLQ-BR23* quality of life questionnaire breast cancer.

**P < 0.01.

## Discussion

The present study analyzed the responsiveness of US biomarkers of muscular and fat echotexture after an exercise intervention and their relationship with PRO in MBC patients. As far as the authors are aware, this is the first study to analyze these outcomes after an exercise intervention in this oncological population. As a result, some US outcomes showed significant statistical changes after the intervention. As the main finding, QoL measured by QLQ-BR23 and CRF measured by the PFS-R scale correlated significantly with some US outcomes. In addition, the regression analysis showed that some US Energy and Textural Correlation from Q Muscle Non-Contraction explained 90% of QLQ-BR23 variance after adjusting by age and weight. Therefore, the hypothesis of this study was fulfilled.

The correlation between QoL and some US outcomes could be because QoL implies a multidimensional construct which includes dimensions such as physical functioning, which is related to muscle function, and fatigue^42^. These relationships between QoL and some US outcomes are quite valuable, as QoL is a key outcome that can even predict mortality in the cancer population^43^. In this regard, muscle mass has also been associated with mortality^44^.

Regarding responsiveness, only 3 out of 80 US analyzed variables (Muscle Non-Contraction Contrast and Subcutaneous Fat Non-Contraction Entropy for Q, and the Difference of Homogeneity between non-contraction and contraction images of the Subcutaneous Fat for BB) reported statistically significant changes after exercise intervention (Table 3). However, PRO analysis did not report any significant change, although QoL and CRF tended to decrease. In oncology, these variables benefit the most from exercise interventions for cancer survivors^45,46^. However, exercise interventions in patients with current cancer diseases such as bone metastases are mainly aimed at ameliorating deterioration^47^. Oncological treatment and cancer could be another factor influencing changes in muscle mass, as they induce muscular adaptations that may counteract those induced by exercise training^48^. As described in Table 1, all patients from the present study received chemotherapy; four were under chemo treatment during the exercise intervention. Otherwise, prior literature suggests that, resistance exercise interventions in patients undergoing cancer treatment reduce body fat but only maintain muscle mass despite gaining strength^49^. Therefore, significant changes seen only in 3 out of 80 US variables may be due to the ameliorated worsening of symptoms and changes in body composition.

Concerning the regression model, Q Muscle Non-Conn Energy and the Q Muscle Non-Contraction Textural Correlation, after adjusting by age and weight, explained 90% of QLQ-BR23 variance (R^2^ = 0.90, *P* < 0.01). A previous study in this population found that an architecture US outcome from BB muscle after adjusting by age and weight explained 70% of QLQ-BR 23 variance^26^. Comparing both results shows that muscular echotexture from Q muscle explains better QoL in MBC patients than muscular architecture from BB. However, further studies with a larger number of patients need to compare muscular architecture and echotexture from BB and Q in this population and their implication in QoL in this oncology population.

The second-order echotexture variables analyzed in this study were first proposed to provide additional information through gray-level patterns in healthy^50^ and amyotrophic lateral sclerosis population^28^, but they have not been previously studied in the cancer population. Some of these second-order parameters have shown good discrimination capacity and correlation with clinical variables, and a combination of both new (GLCM and EV) and traditional (EI) quantitative parameters seems to be a promising biomarker^28^. This fact may increase, for example, the ability to detect lower motor neuron impairment (28). In addition, a pilot study suggested that echotexture variables could be used to monitor disease progression by measuring muscle deterioration through 20 weeks in 13 patients with amyotrophic lateral sclerosis due to the loss of motor neuron^51^. In oncology patients, chemotherapy is supposed to generate oxidative stress to normal tissue, negatively impacting in skeletal muscles and fatigue^52^, and causes morphological changes such as reductions in myofiber size and mitochondria-related damage among neurogenic alterations^53^. At the same time, BC patients are likely to be physically inactive during cancer treatment^54^, which drives to shift of muscle fibers with a transition to a more glycolytic phenotype. Therefore, anticancer treatment toxicity and deconditioning contribute to changes in body composition and CRF^55,56^. As muscle heterogeneity increased in 20 weeks in amyotrophic lateral sclerosis due to the neoformation of non-contractile tissue through denervation^51^, significant changes were observed in this study. In contrast, Homogeneity and Entropy of muscle and subcutaneous fat could report changes in these tissues.

Results from the present study are of special relevance. Firstly, looking at intragroup differences, some US outcomes revealed good responsiveness (Table 2), while PRO did not reach significant differences. In light of these results, US biomarkers are shown promising outcomes for measuring changes after exercise interventions. Secondly, while exercise interventions can face muscle wasting in cancer patients^57^, current research recommends appropriately selecting assessment techniques to measure the effects of interventions^58^. Given results found in responsiveness, proposed US outcomes could be integrated as part of a comprehensive assessment to produce evidence supporting the role of exercise in this population. Lately, it should be noted that these US biomarkers were correlated to QoL and explained its variance. This is of great interest, as QoL predicts mortality in BC older patients^43^. Future longitudinal research should include these US outcomes to study their possible contribution to predictive models for prognosis or mortality purposes. In addition, future research should include presented US outcomes in randomized controlled trials to study the differences between an intervention and a control group.

## Conclusions

The current study analyzed US echotexture biomarkers´s responsiveness to a 12-week exercise intervention in MBC patients and its relationship with PRO. Some echotexture biomarkers related to Contrast, Homogeneity and Entropy showed good responsiveness after the exercise intervention. Some muscle and subcutaneous fat variables correlated positively or negatively with QLQ-BR23 and CRF measured by the PFS-R scale. Q Muscle Non-Contraction Energy and Q Muscle Non-Contraction Textural Correlation explained 90% of the variance of QLQ-BR23 once corrected by age and weight. Future research should address proposed US echotexture biomarkers to measure the effect of exercise interventions in MBC patients.

## Data Availability

The datasets used and/or analyzed during the current study are available from the corresponding author upon reasonable request.

## Abbreviations

BB: Biceps brachii and brachialis
MBC: Metastatic breast cancer
PFS-R: Piper fatigue scale-revised
PRO: Patient-reported outcomes
Q: Quadriceps
QLQ-BR23: Quality of Life Questionnaire Breast Cancer
QoL: Quality of life
US: Ultrasound imaging
